# Future Energy Breakthroughs: Implications for the Hydrocarbon Economies of the Arabian Gulf

**DOI:** 10.1002/gch2.202400151

**Published:** 2024-10-31

**Authors:** Logan Cochrane, Dhabia Al Mohannadi, Sa'd Shannak, Yoshihide Wada, Esra Al Eisa, Mohamad Hejazi

**Affiliations:** ^1^ College of Public Policy Hamad Bin Khalifa University Education City Doha 34110 Qatar; ^2^ Texas A&M University Qatar Education City Doha Qatar; ^3^ College of Science and Engineering Hamad Bin Khalifa University Education City Doha Qatar; ^4^ Qatar Environment and Energy Research Institute Education City Doha 323125 Qatar; ^5^ Biological and Environmental Science and Engineering Division King Abdullah University of Science and Technology Thuwal 23955 Saudi Arabia; ^6^ Industrial and Management Systems Engineering Department Kuwait University 5969 Safat Kuwait 13060 Kuwait; ^7^ King Abdullah Petroleum Studies and Research Center Riyadh 372844 Saudi Arabia

**Keywords:** economics, energy, Planning and design, Policies, politics and governance

## Abstract

Countries in the Arabian Gulf are reliant upon hydrocarbons for revenues, exports, industries and funding services. It is largely assumed that the global energy transition will be gradual, as reflected in planning and strategy documents. However, energy breakthroughs can change the global energy system. This Perspective article seeks to provoke a discussion about potential energy breakthroughs, the plausibility of their rapid expansion at scale, and the implications they may have for the hydrocarbon economies in the Arabian Gulf. Based upon feasibility, scalability, and adoption potential energy breakthroughs are outlined, their probability are assessed, and potential impacts on the hydrocarbon economics of the region are evaluated. The calls to actions are concluded with aim to support the region to be better prepared to track breakthroughs, and be proactively engaged to benefit from them. These include: 1) annual regional research‐policy interface meetings, 2) tailored research and development funding that fosters regional collaboration, 3) investment into breakthrough technologies and energy transition inputs, and 4) seeking synergy in economic diversification regionally to avoid duplication and counterproductive competition.

## Graphical abstract

Potential breakthroughs in energy supply and assessment of the breakthrough likelihood within the next decade. These breakthroughs will create opportunities and challenges for the Arabian Gulf countries, where hydrocarbon resources are focal for government revenues, economic activity and exports.

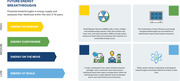

## 1 Introduction

The Gulf Cooperation Council (GCC; Bahrain, Kuwait, Oman, Qatar, Saudi Arabia, United Arab Emirates) member states hold a significant global share of proven hydrocarbon reserves; approximately a third of crude reserves^[^ ^1^ ^]^ and nearly a quarter of natural gas reserves.^[^ ^2^ ^]^ These hydrocarbon resources are critical for the economies of the GCC, accounting for approximately 40% of gross domestic product (GDP; ranging between 20% and 50% for member states) and contributes about half of recent GDP increases (ranging between 0% and 70% for member states.^[^ ^3^ ^]^ Hydrocarbons and related commodities account for a majority of government revenues (ranging from 40% to 90% amongst member states) and export value (ranging between 50% and 90% amongst member states.^[^ ^4^ ^]^ For the GCC, this specifically means oil, with the exception of Qatar where its main hydrocarbon is natural gas and export liquified natural gas.^[^ ^5^ ^]^ These financial flows are critical inputs for basic services, such as electric and water provision.^[^ ^6^ ^]^ Resource revenues are utilized by GCC governments to serve growing populations,^[^ ^7^ ^]^ and as enablers for the region's own economic diversification efforts ^[^ ^8^ ^]^ and are key sources of domestic power generation (primarily natural gas, with the exception of Saudi Arabia.^[^ ^9^ ^]^

Long‐term energy planning often assumes that the global energy transition will occur gradually, which is also seen in GCC national planning documents.^[^ ^10^, ^11^, ^12^ ^]^ However, mitigating climate change and achieving the Paris Agreement will require a rapid energy transition.^[^ ^13^ ^]^ If technology, political will, regulation, and investment align, transition can potentially occur rapidly in certain regions, as past transition experience show is possible. France, for example, had less than 10% of its electricity production from nuclear in 1977 and in less than a decade, in 1986, it surpassed 70%.^[^ ^14^ ^]^ The development of shale extraction technologies emerged out of a breakthrough and enabled the United States to quickly become the world's largest oil and gas producer, changing the hydrocarbon market. Solar and electric vehicles, in some regions like China, have been rapidly adopted at scale. These shifts did not entirely transform the global energy system, but each have significant impacts.

This Perspective explores potential energy breakthroughs, the plausibility of their rapid expansion at scale, and the potential positive and negative implications these breakthroughs may have for the GCC. In the following section, we briefly situate potential energy breakthroughs and their medium‐term likelihood (the coming decade), which is informed by feasibility, scalability, and adoption potential. We conclude with a call to action for the GCC region regarding 1) annual regional research‐policy interface meetings, 2) tailored research and development funding that fosters regional collaboration, 3) investment into breakthrough technologies and energy transition inputs, and 4) seeking synergy in economic diversification regionally to avoid duplication and counterproductive competition.

## 2 The Breakthroughs

**Figure 1:**
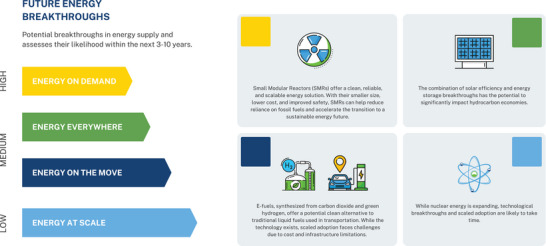
Future energy breakthroughs and likelihood in coming decade.

The following briefly describes potential supply‐side breakthroughs and assesses their likelihood in the medium term (coming decade) based on feasibility, scalability, and adoption potential (see **Figure** **1**). These breakthroughs are not mutually exclusive; in fact we anticipate developments to occur in parallel and heterogeneously on a global scale. While this Perspective analyzes potential changes to the energy sector, the supply‐side focus does not analyze energy efficiencies (we recognize breakthroughs in other areas of the energy system may support the decarbonization and emission reduction transition).

1. Energy on Demand: Small Modular Reactors (SMRs) could be rapidly scaled through industrial production with comparatively low cost while also offering improved safety features compared to nuclear plants. Low capital requirements, zero direct emissions, scalability and its ability to act as a reliable baseload energy position SMRs to bring energy where it is needed, in the amount needed. The Nuclear Energy Agency SMR dashboard reports a diverse pipeline of developments, with several SMRs expected to be commercialized before 2030.^[^ ^15^ ^]^ If SMRs were scaled globally, this would impact oil and gas exports (e.g., natural gas used for power generation and/or residential use could be substantially reduced or eliminated) as systems are electrified. Access to cheap, clean energy may enable other transitions, such as a more rapid transition to electric vehicles, further reducing oil demand. While there would be a market for hydrocarbons even if SMRs were widely adopted, globally and/or in the GCC, the demand trend may shift directions from positive to negative. Given that the technology for SMRs is already in place, what is primarily needed for a breakthrough is an investment, manufacturing and regulatory breakthroughs, which may result in heterogeneous global adoption. We view the likelihood of heterogeneous SMR scaling in decade as medium to high.
2. Energy Everywhere: Solar energy adoption has expanded globally. Breakthroughs may make this energy source even more efficient (e.g., developments in technology such as perovskite technology and new expansions such as floating solar). Transformative levels of adoption of solar, and other renewable energy sources, require parallel breakthroughs in energy storage (e.g., producing green hydrogen using renewables, creating a transportable clean replacement fuel for oil and gas; renewable sources used to produce synthetic natural gas, such as via electrolysis). Energy storage has made progress (e.g., lithium batteries), further advancements are required to scale. A breakthrough (e.g., sodium) could cause a shift in the liquid fuels sector and a decline in demands on the GCC hydrocarbon export. Thus, the combination of these breakthroughs (solar efficiency and energy storage) has the potential to disrupt or reshape the hydrocarbon economies of the GCC significantly. We view solar efficiency breakthroughs as medium likelihood in the next decade.^[^ ^16^ ^]^ In the case of a breakthrough, adoption would be heterogeneous due to varied global financial ability to scale, and given experience to‐date may also be slower to adopt in the GCC.^[^ ^17^ ^]^ We view commercially viable breakthroughs in energy storage of low likelihood in the next decade.^[^ ^18^ ^]^ In the case of a breakthrough, we expect scaled adoption to have a low to medium likelihood, less due to technology per se but due additional implementation requirements (e.g., infrastructure for hydrogen, cost comparisons to other hydrogen sources during when implemented) as well as limitations on the required inputs (e.g., cobalt, nickel, copper; ^[^ ^18^ ^]^). The combination of these two breakthroughs occurring simultaneously in the near term is therefore unlikely.
3. Energy on the Move: One of the main uses of oil is for the transportation sector (e.g., aviation, shipping, vehicles). Electro‐fuels (E‐Fuels) are liquid fuels synthesized from carbon dioxide and green hydrogen. With the increased demand for carbon capture, the captured carbon dioxide will end up either in geological storage or utilized. E‐fuels could be one of the utilization options that can be carbon neutral (or even carbon negative if derived from direct air capture). The technologies (e.g., hydrogen electrolysis) and processes (Ficher‐Tropsch synthesis) are available and E‐Fuels could be integrated into some existing infrastructure as a substitute (e.g., e‐methanol, e‐diesel, e‐gasoline). With this breakthrough, countries with access to consistent renewable energy would be the new suppliers of the world's liquid fuels as opposed to the GCC. The startup space has been active in the e‐fuels sector, with some companies reaching unicorn status and international energy companies listing the technology as part of their decarbonization strategies. Despite the rapid expansion that has occurred, scaled adoption remains years away due to the lack of large‐scale implementation and cost barriers.^[^ ^19^ ^]^ We assess this breakthrough as having a low likelihood to be scaled sufficiently to impact the energy system in the next decade (breakthroughs that occur may be limited to specific sectors.^[^ ^20^ ^]^
4. Energy at Scale: The breakthrough of energy at scale could be new energy production technologies, such as nuclear fission and new forms of nuclear that address current barriers (e.g., thorium reactors and molten salt reactors that are less water intensive for arid environments) or even more significant expansions of nuclear reactors. Nuclear reactors are currently being expanded (as of 2022, 411 were operational, 58 under construction, 27 in suspended operation, and approximately 110 planned). The majority of planned reactors are in Asia, which is the primary market for GCC hydrocarbons.^[^ ^21^ ^]^ At COP28, more than twenty nations pledged to continue the nuclear expansion, marking a (re)turn to nuclear.^[^ ^22^ ^]^ However, the scaling process of nuclear reactors would be slower due to construction time and therefore the short‐ and medium‐term risks comparatively low. For example, the development of the nuclear power plant in the UAE required long‐term planning (contracting began in 2009, construction started in 2011, commercial operations started in 2021). As a first for the region, future developments may be faster; however, no other plants in the GCC have begun this process. Due to the timelines of either new technology or scaled nuclear reactors, we view the likelihood of significant breakthroughs in these domains impact the energy sector within the coming decade as very low.

## 3 The Implication for GCC Hydrocarbon Economies: Challenges and Opportunities

The economies of the GCC rely upon hydrocarbon in multiple ways. These dependencies also exist for domestic needs. Consider the supply of water, for which the GCC relies on non‐conventional sources like thermal and membrane desalination.^[^ ^23^ ^]^ Transitioning to renewables is challenging in this energy‐intensive process. Currently, only 1% of desalination in the Middle East is powered by renewable energy sources,^[^ ^24^, ^25^ ^]^ highlighting both the challenge and the opportunities of energy breakthroughs. SMRs, for example, may provide the scalability and energy intensity required to continue to provide water, while also reducing domestic hydrocarbon consumption and GHG emissions, but may raise a new issue of ionizing radiation accompanied with all associated risks. The same is the case in the GCC for cooling demands, which rely on domestic hydrocarbon consumption for required energy production. In the energy on demand scenario, energy systems become more distributed, requiring system redesign and significant investment and policy reform, without which GCC countries may not be able to capitalize upon emerging breakthroughs.^[^ ^26^, ^27^, ^28^ ^]^

Breakthroughs in E‐Fuels may impact global hydrocarbon demand (as earlier noted; not an end to the sector, but a transition from growing year‐on‐year demand to declining) while breakthroughs in energy production may impact gas demand. The GCC has the potential to navigate these transitions in ways that benefit their economies (e.g., focusing on value‐added sectors and developing manufacturing that rely on hydrocarbon inputs). As an investor or forerunner, the GCC could also build on prior efforts such as in carbon capture utilization and storage and clean hydrogen production to be a leader in adopting breakthroughs, which could be utilized in energy‐intensive sectors (e.g., desalination, cooling, manufacturing). However, if these challenges are not navigated proactively, and alternative economic pathways are not supported with enabling policy as well as research and development, the prosperity of the region may be stalled in the long run due to declining revenues (with risks not only of demand but also of price declines of hydrocarbons). As was experienced during the COVID19 pandemic, declining demand and/or prices places significant financial strain on the provision of essential services.

The GCC has a wealth of experience that it can draw upon in navigating potential disruptions to planning based on conventional wisdom. For example, the region had to grapple with food supply disruptions due to conflict and global health emergencies, particularly challenging for a net food importing group of countries. Sovereign wealth funds have been utilized to mitigate economic disruptions in the GCC, building domestic strategic foresight capacity. In the energy sector, the above‐mentioned development of nuclear power in the UAE is another effort of diversification, which took long‐term planning (including in the education sector, to ensure the availability of the required skilled talent). Within the last decade, the GCC has undergone a wide range of changes in the energy spheres (e.g., reforming energy prices) as well as economic restructuring (e.g., introducing value‐added tax, fiscal balancing over commodity cycles). The social and political implications of these significant changes provide insight for the GCC on how to successfully navigate substantive change (e.g., enabling new industries to navigate potential labor market disruptions; phasing transitions for industries to maintain competitiveness; targeting subsidies to ensure access to energy for all member of society.^[^ ^29^, ^30^ ^]^ Regional advancements in renewable energy inputs, such as large solar projects in Qatar and Saudi Arabia, seek to diversify domestic energy capacity away from hydrocarbons and optimize available resources (see, for example: ^[^ ^31^, ^32^ ^]^). Efforts to ensure the hydrocarbon industry retains market share may mean advancing research and development in carbon capture technologies, reducing the carbon footprint of products. Investments in ammonia and hydrogen throughout the GCC are notable, such as Qatar opening the world's largest ammonia plant in 2026 and large hydrogen projects underway in Oman.

## 4 Conclusion: Call to Action

Given the size of the GCC (population, economy, industrial and manufacturing sector, et cetera) we argue that the challenges require regional cooperation and collaboration, and we have therefore emphasized the role of the GCC Secretariat. This builds on the strength of regional collaboration in wide ranging areas from common regulation (e.g., GCC Standardization Organization) to broader regional climate and energy collaborations.^[^ ^33^ ^]^

1. The GCC Secretariat General should host annual research‐policy interface meetings to monitor and track regional developments, with specific sessions dedicated to breakthroughs, disruptions and risks. This should include regular progress notes to track progress across the GCC. Based on the emerging evidence, governments need to regularly conduct assessments to support proactive decision making that utilizes strategic management tools, such as foresight and objective planning.
2. Research and development (R&D) funding in the GCC region remains lower than global averages, however the region has been witnessing increased R&D funding especially in clean energy R&D as part of broader diversification strategies such as Saudi Vision 2030 and UAE Vision 2021 (World Bank, 2022). Building on this momentum, R&D in all member countries can be used to foster innovations, tailor and adapt solutions, support the monitoring of developments, and be actively engaged in the annual research‐policy interface run by the GCC Secretariat General. In addition, to foster research collaboration, granting agencies can require that certain tracks of funding require collaboration from a research partner based in at least one other GCC member state. Creating these enabling opportunities will encourage research to break national silos and facilitate regional exchange and learning. This could include investing in regional innovation hubs for energy solutions that capitalize on the GCC strength and know‐how while leading on future markets and trends in the energy sector.
3. Sovereign wealth funds (SWFs; such as Saudi's Public Investment Fund, UAE's Abu Dhabi Investment Authority and the Investment Corporation of Dubai, Qatar's Investment Authority, Kuwait's Investment Authority, et cetera) collectively manage trillions of dollars. These could further invest in strategic breakthrough technologies, start‐ups and supply chains. This would enable advantages in knowledge and access. Energy breakthroughs also present opportunities for the GCC to lead in future global markets. For example, the electrification of energy systems combined with the increased demand of energy throughout Africa and Asia will require new inputs for infrastructure expansion (e.g., copper) and energy generation (e.g., uranium). The markets for such commodities are relatively small, while the GCC governments have large SWFs that could be utilized to gain market share in the new energy sector. Saudi Arabia has made notable moves in this direction, with the London Metals Exchange adding a delivery point in Jeddah and its investments in mining (e.g., US2.6 billion with Vale SA; ^[^ ^34^ ^]^).
4. All countries in the GCC are seeking to diversify their economies and transition from a hydrocarbon‐based economy to a knowledge‐based economy given the expected global energy shifts. A risk is that in implementing this transition, GCC member states create duplication and unhealthy state‐supported counterproductive competition. High‐level engagement can find opportunities for synergy and collaboration (e.g., one member state develops an EV manufacturing sector, another a batteries sector, another e‐fuels hub whereby all benefit from regional developments) such that the state‐supported knowledge‐economy transitions are done in ways that complement regional activities.

## Conflict of Interest

The authors declare no conflict of interest.

## References

[1] M. A. Al Suwailem , A. Aldayel , in, Crude Oil Reserves Metrics of GCC Members, King Abdullah Petroleum Studies and Research Center, Mecca 2020.

[2] N. Inuwa , S. Adamu , M. B. Sani , A. M. Saidu , Biophys. Econom. Sustainability 2022, 7, 13.

[3] T. Cullinan , G. Filocca , in, GCC Economic Activity Held Back By Its Hydrocarbon‐Heavy Economic Structure And OPEC‐Related Production Cuts, S&P Global, New York 2020.

[4] N. Kabbani , N. B. Mimoune , in, Economic diversification in the Gulf: Time to redouble efforts, Brookings Institution, Washington, DC 2021.

[5] EIA, Natural Gas – Annual. U.S. Energy Information Administration, in: U.S.E.I. Administration (Ed.), Independent Statistics and Analysis, U.S. Department of Energy, Washington, DC 2024.

[6] L. Cochrane , R. Al‐Hababi , Sustainable Qatar: Social, Political and Environmental Perspectives, Springer Nature, Berlin 2023.

[7] A. Ali , L. Cochrane , Compar. Migration Studies 2024, 12, 16.

[8] W. Bank , in, Economic Diversification Efforts Paying Off in GCC Region but More Reforms Needed, The World Bank Group, Washington, DC 2023.

[9] S. Brown , D. Jones , in, European Electricity Review, Ember, England 2024, 2024.

[10] A. Al‐Sarihi , in, The GCC and the road to net zero, Middle East Institute, Washington, DC 2023.

[11] Saudi vision, Saudi Green Initiative Project, 2021.

[12] IAEA , Energy, Electricity and Nuclear Power Estimates for the Period up to 2050, International Atomic Energy Agency, Vienna, Austria 2023.

[13] UNFCCC , Global Stocktake, Bon, Germany 2023.

[14] World Bank , Electricity production from nuclear sources (% of total) – France, The World Bank Group 2024.

[15] OECD/NEA , in The NEA Small Modular Reactor Dashboard, Paris 2023.

[16] N. Li , Z. Shi , C. Fei , H. Jiao , M. Li , H. Gu , S. P. Harvey , Y. Dong , M. C. Beard , J. Huang , Nat. Energy 2024.

[17] A. Farahat , A. H. Labban , A.‐W. S. Mashat , H. M. Hasanean , H. D. Kambezidis , Clean Technol. 2024, 6, 700.

[18] T. Mu , Z. Wang , N. Yao , M. Zhang , M. Bai , Z. Wang , X. Wang , X. Cai , Y. Ma , J. Energy Storage 2023, 69, 107917.

[19] A. Boretti , Int. J. Hydrogen Energy 2024, 79, 258.

[20] N. Detsios , S. Theodoraki , L. Maragoudaki , K. Atsonios , P. Grammelis , N. G. Orfanoudakis , Energies 2023, 16, 1904.

[21] WNA , Plans For New Reactors Worldwide, in: World Nuclear Association, world‐nuclear.org, London, UK 2024.

[22] J. Donovan , in, Nuclear Energy Makes History as Final COP28 Agreement Calls for Faster Deployment, International Atomic Energy Agency, Vienna, Austria 2023.

[23] E. Aleisa , Desalination 2024, 586, 117827.

[24] N. Ghaffour , S. Lattemann , T. Missimer , K. C. Ng , S. Sinha , G. Amy , Appl. Energ. 2014, 136, 1155.

[25] A. Mahmoudi , M. Bostani , S. Rashidi , M. S. Valipour , Renewable Sustainable Energy Rev. 2023, 184, 113543.

[26] M. C. Brisbois , Global Trans. 2020, 2, 16.

[27] A.d. Giovanni , B. Warren , in Can Decentralized Energy Get Good Enough, Fast Enough?, Ernst & Young Global Limited, London, UK 2022.

[28] Y. Sun , P. Gao , A. Razzaq , Renewable Energy 2023, 206, 1064.

[29] J. Nordenson , J. Arabian Studies 2020, 10, 139.

[30] S. Olver‐Ellis , in Building the new Kuwait: Vision 2035 and the Challenge of Diversification, L.R.O.D.o. Economics (Ed.) LSE Middle East Centre Paper Series, London School of Economics and Political Science, London, UK 2020.

[31] EIU , in Saudi Arabia Launches World's Largest Solar‐Power Plant, UK 2023.

[32] P. Pouyanné , A.l Kharsaah , in A Pioneering Solar Power Plant in Qatar 2024.

[33] OAPEC , in Organization of Arab Petroleum Exporting Countries 2024.

[34] Bloomberg , Saudi's $2.6 Billion Deal Is Big Oil's Latest Mining Push, Bloomberg 2023.

