# Correction to “Future Energy Breakthroughs–Implications for the Hydrocarbon Economies of the Arabian Gulf”

**DOI:** 10.1002/gch2.70011

**Published:** 2025-06-13

**Authors:** Logan Cochrane, Dhabia Al Mohannadi, Sa'd Shannak, Yoshihide Wada, Esra Al Eisa, Mohamad Hejazi

*Global Challenges*, **2024**, *8*, 2400151

DOI: 10.1002/gch2.202400151

When we submitted the article, we listed the funder in the EM system, but that does not appear in the article itself.

Please add funding acknowledgement:

Funding: The research reported in this publication was supported by the Qatar Research Development and Innovation Council project [NPRP14C‐0920‐210017]. The content is solely the responsibility of the authors and does not necessarily represent the official views of the Qatar Research Development and Innovation Council.

